# Socioeconomic Inequalities in the HIV Testing during Antenatal Care in Vietnamese Women

**DOI:** 10.3390/ijerph16183240

**Published:** 2019-09-04

**Authors:** Dinh-Toi Chu, Hoang-Long Vo, Dang-Khoa Tran, Hao Nguyen Si Anh, Long Bao Hoang, Phong Tran Nhu, Khanh Nguyen Ngoc, Trang Thu Nguyen, Quyet Pham Van, Nguyen Le Bao Tien, Vo Van Thanh, Vu Thi Nga, Thuy Luu Quang, Le Bui Minh, Van Huy Pham

**Affiliations:** 1 Faculty of Biology, Hanoi National University of Education, Hanoi 100000, Vietnam; 2 Institute for Preventive Medicine and Public Health, Hanoi Medical University, Hanoi 100000, Vietnam (H.-L.V.) (H.N.S.A.) (K.N.N.) (T.T.N.) (Q.P.V.); 3 Department of Anatomy, University of Medicine Pham Ngoc Thach, Ho Chi Minh City 700000, Vietnam; 4 Institute of Gastroenterology and Hepatology, Hanoi 100000, Vietnam; 5 Public Health Department, Nursing Faculty, Dai Nam University, Hanoi 100000, Vietnam; 6 Institute of Orthopaedics and Trauma Surgery, Viet Duc Hospital, Hanoi 100000, Vietnam (N.L.B.T.) (V.V.T.); 7 Department of Surgery, Hanoi Medical University, Hanoi 100000, Vietnam; 8 Institute for Research and Development, Duy Tan University, Danang 550000, Vietnam; 9 Center for Anesthesia and Surgical Intensive Care, Viet Duc Hospital, Hanoi 100000, Vietnam; 10 NTT Hi-tech Institute, Nguyen Tat Thanh University, 300A Nguyen Tat Thanh St., Ward 13, District 4, Ho Chi Minh City 700000, Vietnam; 11 AI Lab, Faculty of Information Technology, Ton Duc Thang University, Ho Chi Minh City 700000, Vietnam

**Keywords:** socioeconomic inequalities, ethnicity, HIV testing, pregnancy, Vietnamese women

## Abstract

Although HIV (human immunodeficiency virus) testing for all women has been promoted by Vietnam’s Ministry of Health since 2000, test acceptance rates in this country were reported to be less than 30% in the community. This country has been facing the barriers to approach the national services towards transmission prevention from mother to child including HIV testing during antenatal care (ANC) towards mothers. Here, we aim to assess the socioeconomic inequalities in HIV testing during ANC among Vietnamese women. This study used available data from the Vietnam Multiple Indicator Cluster Survey 2014. Overall, the prevalence of HIV testing during antenatal care was 30% and the concentrate index (CCI) was 0.1926. There was significant inequality between women classified as poor and rich, and when stratified by social characteristics, inequality was found in women aged 15–49 years (CCI: 0.4), living in rural areas (CCI: 0.3), belonging to ethnic minorities (CCI: 0.5) and having primary or less education (CCI: 0.4). In the multivariate logistic regression analysis, ethnicity and socioeconomic status were significant factors associated with HIV testing during ANC. We found the prevalence of HIV testing during ANC was low, and its inequalities were associated with age, living area, ethnicity, education, and economic status.

## 1. Introduction

The human immunodeficiency virus (HIV) epidemic is one of the global priority health concerns with existing difficulties in control and prevention. According to the report of The Joint United Nations Program on HIV and AIDS (UNAIDS), an estimated 5.2 million people with HIV were in the Asia-Pacific region in 2017 [1]. During the 2010–2017 period, the Asia-Pacific region has seen a 14% decline in new HIV infections (NHI) [2], and particularly, a significant 33% decline in NHI in children [3]. In the Asia-Pacific region, the prevalence of pregnant women living with HIV was 1%–2% [4,5,6], lower than the figure for sub-Saharan Africa (5%–37%) [7,8]. The HIV prevention programmes, such as prevention of parent to child transmission (PPTCT), have been considered as a crucial part of reducing new HIV infection. PPTCT significantly reduced the HIV infection rate among newborn infants [9] and is now available across the Asia-Pacific region [10]. Between 2010 and 2018, PPTCT helped prevent around 1.4 million children worldwide from HIV infection [11].

HIV testing during pregnancy is an effective primary strategy as well as an entry point for PPTCT [12]. Despite significant progress towards scaling up PPTCT in many countries, previous studies reported low rates of HIV testing during pregnancy [6,13,14]. The HIV testings’ reported barriers during pregnancy included socio-demographic status [15,16,17], mothers’ knowledge of the contents of mother-to-child transmission as well as PPTCT [18,19,20], and attitude towards HIV/AIDS [14,21,22].

Vietnam, a developing country in Southeast Asia with three-quarters of the population living in rural areas, has experienced a high burden of HIV in more than two decades after Doi Moi (Doi Moi (English: Renovation) is the name given to the economic reforms initiated in Vietnam in 1986 with the goal of creating a “socialist-oriented market economy”. The term ‘Doi Moi’ itself is a general term with wide use in the Vietnamese language.) [23]. The prevalence of HIV-positive pregnant women was 0.37%, but the figure was underestimated [24]. Early diagnosis of HIV infection through HIV testing during ANC is needed to reduce HIV infections for newborns and for subsequent HIV treatment, care, and support services for HIV-positive women [25]. However, according to the World Health Organization (WHO), Vietnam meets the challenges in initial diagnosis of HIV infection for pregnant women. Although the Ministry of Health in Vietnam has advanced to HIV testing for all women since 2000, the rates of test acceptance reported in this country were less than 30% in the community [22,26]. Furthermore, Vietnam’s government has introduced a campaign against HIV/AIDS, including the goals of 90% of pregnant women receiving HIV counselling and testing and 100% of those who are HIV-positive being provided with preventive interventions for HIV-positive individuals [4]. However, the national economic development’s benefits in Vietnam have not been a balanced distribution, with many of these being put into more advantaged groups. This situation has made the inequalities become worse in the PPTCT services which were integrated into the antenatal services at different levels of healthcare facilities in Vietnam. There is currently no nationally representative study addressing inequalities-based multiple socioeconomic dimensions in HIV testing during ANC in Vietnamese women. Hence, available data of the Vietnam Multiple Indicator Cluster Survey (MICS) 2014 was used with the aim of assessing socioeconomic inequalities (SEI) in HIV testing during ANC among Vietnamese women aged 15–49 years who experienced a live birth (LB) in the last two years.

## 2. Methods

### 2.1. Source Data

We analysed the data from the 2014 round of MICS in Vietnam to examine socioeconomic inequalities in HIV testing during ANC among 15–49-year-old women (childbearing age) with a LB in the last two years [27]. The United Nations Children’s Fund (UNICEF) designed MICS to gather internationally comparable data of women and children. The Vietnam MICS was carried out with financial and technical support of the General Statistics Office of Vietnam, UNICEF, and the United Nations Population Fund. The sample for the Vietnam MICS 2014 was designed to provide estimates for a large number of indicators on the situation of children and women at the national level, for urban and rural areas of Vietnam as well as six geographic regions in the country, the Red River Delta, Northern Midlands and Mountainous area, North Central and Central Coastal area, Central Highlands, and South East and Mekong River Delta. The sampling frame used for the selection of sample clusters for the Vietnam MICS 2014 was based on a 15 percent sample of enumeration areas used for the long form questionnaires of the Population and Housing Census 2009 (PHC2009). In the MICS 2014 round, a multi-stage, stratified cluster sampling approach was applied to select the sample. Two sampling stages were done for region, urban, and rural areas. In each, region, urban, and rural areas were identified as the main sampling strata. In each stratum, a specified number of census enumeration areas were selected systematically with probability proportional to size. The MICS collected a list of households and then a sample of households was drawn in each area. The sample was stratified by regions and areas. The total number of households was 10,018 households in the MICS 2014. Three sets of questionnaires were used in the survey to collect information on household, women aged 15 to 49 years, and children under 5 years old. The contents of the MICS have been presented in detail elsewhere [27].

In the present study, we extracted data for women aged 15 to 49 years, who had had a live birth within the last 2 years at the time of being interviewed, from the dataset of the MICS 2014 round. Then, we focused on one of the reported indicators in the HIV testing section from the MICS 2014 as HIV testing during ANC to analyse for this paper. A total of 1484 women aged 15 to 49 years having a LB within the last 2 years were included in the MICS 2014.

### 2.2. Independent and Explanatory Variables

#### 2.2.1. Independent Variable

The independent variable was HIV testing during ANC among 15–49-year-old Vietnamese women who had had a LB in the last two years (a binary variable). According to the MICS report, HIV testing during ANC was defined as a woman in the antenatal period was offered and accepted HIV testing and knew their results [27].

#### 2.2.2. Selected Explanatory Variables

Selected explanatory variables included age group (15–19, 20–34, and 35–49), education level (primary or less, lower secondary, and upper secondary and tertiary), living area (urban and rural), ethnicity (Kinh and minority), and wealth index quintiles. Household assets of all individuals were taken into account as a measure of economic status, which was estimated with principal component analysis (PCA) [28]. The wealth asset index was constructed by principal component analysis using information on the ownership of consumer goods, dwelling characteristics, water and sanitation, and other characteristics that are related to household wealth in the 2014 MICS dataset [27]. In the Vietnam MICS 2014, the following assets were used in these calculations: radio, television, telephone, refrigerator, table and chair set, fan, computer, air conditioner, gas cooker, electric cooker, washing machine, car or tractor, ship or boat, mobile telephone, bicycle, motorbike, ownership of dwelling, bank account, agricultural land, water surface, forestry land and animals/livestock. First, initial factor scores are calculated for the total sample. Then, separate factor scores are calculated for households in urban and rural areas. Finally, the urban and rural factor scores are regressed on the initial factor scores to obtain the combined, final factor scores for the total sample. This is carried out to minimize the urban bias in the Wealth Index values. The survey household population is then ranked according to the wealth score of the household they live in and is finally divided into five equal parts (quintiles), from lowest (poorest) to highest (richest). The method for estimating the wealth asset index has been described in detail elsewhere [27].

#### 2.2.3. Ethnic Groups

There are 54 ethnic groups of people in Vietnam. The Kinh (Vietnamese people), the majority ethnic group of Vietnam, comprises 86% (over 65 million) of Vietnam’s population. Kinh people live in all provinces and mainly concentrate in about half of the country’s territory, especially in coastal and low-lying areas. Most of the remaining 53 official ethnic groups (ethnic minorities) mainly inhabit the interior mountainous and highlands.

### 2.3. Measurement of Socioeconomic Inequality

#### 2.3.1. Concentration Index

The ‘concentration index’ (CCI) measures inequality in one variable over the distribution of another and is applied to the measurement of socioeconomic-related inequality in health [29,30,31]. In this study, the CCI was used to measure the degree of SEI in HIV testing during ANC among Vietnamese women [30,31]. The CCI value was calculated as follows:

(1) $$CCI=\frac{2}{µ}cov\left(h,r\right)$$

The overall percentage of HIV testing during ANC is *µ*. h was the value for HIV testing of each woman, r is the rank of household socioeconomic status. The CCI value could range between −1 and +1. A CCI value of 0 means completely equal distribution in HIV testing during ANC amongst richer families and those who are poorer. If it is negative, it indicates that the concentration of HIV testing during ANC is higher among those women in poor families, and if it is positive, it indicates that the concentration of HIV testing during ANC is higher among those children in wealthier families [31].

#### 2.3.2. Concentration Curve

The concentration curve (CC) was applied to present the overall degree of SEI in HIV testing during ANC in women in the study. The CC plots the cumulative HIV testing percentage (*y*-axis) compared to the cumulative wealth index percentage from the poorest to the richest (*x*-axis). The diagonal line from the origin reflecting perfect equality is a linear line at 45° (line of equality). The farther the CC lies above the diagonal line, the higher concentration of HIV testing during ANC in poor women, and vice versa if the CC lies below the 45° line. In addition, the farther CC is against the 45° line, the greater the degree of socioeconomic inequality towards HIV testing.

### 2.4. Data Analysis

We described the characteristics of Vietnamese women aged 15–49 and characterized HIV testing during antenatal care among women by different socioeconomic variables. Differences in the proportion of HIV testing during ANC among groups were compared by the Pearson’s chi-squared test. We used the conindex command to compute the overall CCI and all CCIs in subgroups of socioeconomic variables defined by a binary or categorical variable. The conindex command was also applied to provide the results of the statistical test if the CCIs were statistically significantly different from 0, and the difference between the CCIs of two different sets of the study population (using independence two-tailed *t*-test). The multivariate logistic regression was applied to determine the associations with HIV testing during antenatal care among women. All statistical analyses were conducted by Stata® 15 (StataCorp LLC, Lakeway Drive College Station, Texas, USA), using the weighted women-related variables in the dataset. The Coindex package for the Stata® software was employed to estimate the CCI [32].

### 2.5. Research Ethics

The MICS dataset was available to access after requesting to use. All users’ research findings are encouraged to share according to MICS’ mentioned information. The raw data of the 2014 MICS was obtained with the approval to use the data for this study. The MICS dataset is described in detail on UNICEF’s website (http://mics.unicef.org/). All study participants in the MICS had provided informed consent. Individual identifiable information had been removed before the UNICEF made the dataset available to users.

## 3. Results

The proportion of women aged 15–19, 20–34, and 35–49 was 5.4%, 83.5%, and 11.1%, respectively. A majority of the women were Kinh people (83.0%) and lived in rural areas (70.8%). 46.9% of women completed the upper secondary school and tertiary education. Table 1 presents the economic status of the study population.

As was given in Table 2, the overall HIV testing prevalence during ANC among Vietnamese women was 30%. Women belonging to the Kinh ethnic group (34.4%) had more HIV testing than ethnic minorities (8.5%). The proportion of HIV testing during ANC increased with educational levels (from 17% in the primary group to 38.4% in the upper secondary school and tertiary groups). The proportions of HIV testing were 42.2% in urban areas and 25% in rural areas. The prevalence of HIV testing increased with better economic status, from 8.7% in the poorest to 50.1% in the richest. All differences were statistically significant except for age group.

**Figure 1:**
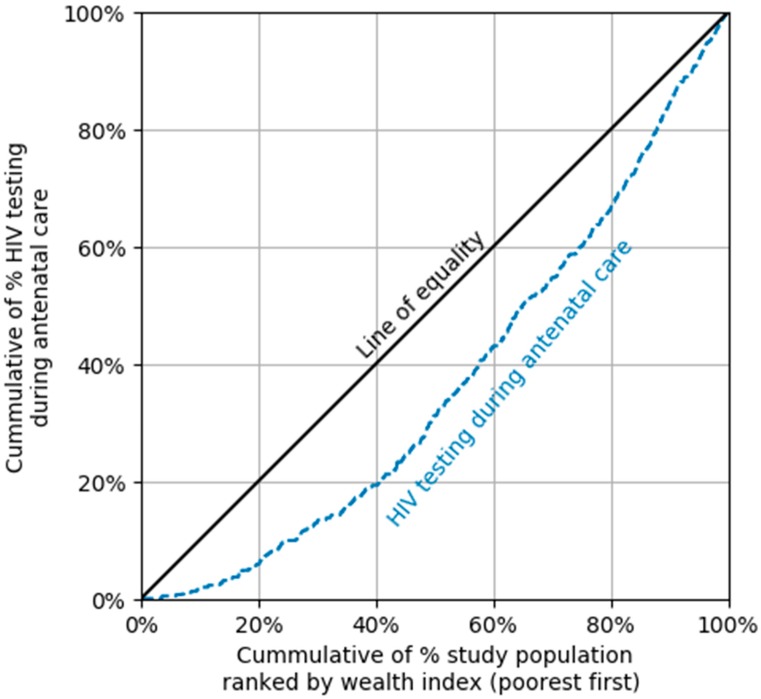
Concentration curve (CC) of HIV testing during antenatal care among women aged 15–49 years who had a live birth in the last two years, by wealth index.

The overall CCI value was 0.3. Inequality in HIV testing during ANC between the poor and the rich existed in people aged 15–19 years (CCI: 0.4), living in rural areas (CCI: 0.3), belonging to ethnic minorities (CCI: 0.5), and having primary or less education (CCI: 0.4) (Table 3). As shown in Figure 1, CC below the equality line indicated that HIV testing during ANC concentrated more in rich women.

In the multivariate logistic regression, Kinh women had significantly higher odds of HIV testing during ANC than those from ethnic minorities (OR 2.52, 95% CI: 1.4–4). The odds of HIV testing during ANC was higher in people with better economic status (Table 4).

## 4. Discussion

HIV testing in the antenatal period is important because a mother tested positive with HIV will immediately receive counselling, antiretroviral therapy, and services such as PPTCT [33,34]. This allows a cost-effective, comprehensive approach to take care of both HIV-positive mothers and their children. Despite the availability of HIV testing to women attending antenatal services at different healthcare facility levels, only 30% of mothers in our study experienced HIV testing during ANC. This prevalence is much lower than the figure reported in a Hanh et al.’s study conducted in several individual provinces in Vietnam (90.3%) [35]. This might be due to the difference in the definition of HIV testing during antenatal care amongst studies. Hanh et al. did not include whether a woman knew the test results in their variable definition. Besides, we considered comparing an Ethiopia study (nationally representative sample size from the 2016 Demographic Health Survey) using the same definition as our study, showing that our study did not much differ from theirs (35.1%) [13].

Previous reports indicated the associations between the users’ characteristics and their circumstances as factors preventing people from HIV testing during ANC [13,36]. Furthermore, available evidences from low-income and low-middle income countries indicated the income was an important barrier that decisively affects HIV testing towards women of childbearing age [13,36,37]. In the present study, the likelihood of HIV testing increased as the wealth index increased from the poorest to the richest groups, specifically, socioeconomic status was significantly associated with women’s attendance of HIV testing. This finding indicated that poverty is a crucial influencing barrier towards HIV testing during ANC. Our result, which reports existing inequality in HIV testing during ANC between richer and poorer groups, is in line with other studies in Vietnam and in other Asian countries [13,36,37].

We found that women who achieved better education had more HIV testing, agreeing with the results in previous studies [13,36,37]. In Vietnam, the proportion of low education (secondary school or below) was higher in people from ethnic minority and/or having low economic status according to the national ethnicity report [38]. Besides, the HIV infection prevalence in several regions can be as high as more than 10 times than the national prevalence, particularly in mountainous and remote areas resided by ethnic minorities [39]. Also, in our study, Kinh ethnicity was associated with higher HIV testing during ANC. Our finding was consistent with other studies’ findings [14,20,35]. The Kinh people live mainly in places with better health services (such as plains and urban areas) and are capable of having better socioeconomic conditions. On the contrary, people from other ethnicities generally have poorer socioeconomic conditions and less approach for health education and services, including HIV testing, treatment, and care [40]. Furthermore, Vietnam has been faced with complex challenges regarding human resources in the health sector. There are not enough healthcare workers working in HIV treatment and care in the rural, remote and mountainous areas (where are common locations of ethnic minorities). Importantly, only one fifth of the districts across Vietnam contain HIV-trained staff [26]. We recommend (i) scaling up the availability of HIV testing in the period of ANC in the rural, remote and mountainous areas, especially in ethnic minority’s residence, and (ii) piloting training programmes for young professionals in these areas.

Our study has several limitations. Firstly, the primary outcome (proportion of women having HIV testing during antenatal care) was calculated based on self-report data. Since the investigators of the original survey did not seek measures to crosscheck self-report data, the data might be subject to recall and reporting bias. Secondly, the MICS data did not allow the users to know maternal refusal or acceptance after them being offered an HIV test during ANC, which could be partly related to socioeconomic status. Beside this, though HIV testing was offered as a part of ANC, many Vietnamese women were tested for the first time only once going into labour, and thus, do not get the full benefit from the PPTCT program. Hence, the rate of HIV testing during ANC could be underestimated, the figure might be lower if this assumption was explored. Thirdly, MICS data did not allow users to assess the knowledge, attitude, and practice of women regarding HIV testing, and stigma and discrimination towards HIV/AIDS. Therefore, we could not exclude the confounding role of these factors in the findings regarding inequality. Fourthly, because the Vietnam MICS in 2014 is the most recent data, socioeconomic inequalities regarding HIV testing during ANC in the last five years could not be accessed. Finally, given the cross-sectional nature of the MICS, no causal relationship can be established.

## 5. Conclusions

In women of childbearing age, the prevalence of HIV testing during antenatal care was low in Vietnam. Inequality in HIV testing was associated with age, living area, ethnicity, education, and economic status. The findings inform the need for a comprehensive strategy targeting women with higher risk of not receiving HIV testing during pregnancy. Not all women are, in fact, offered an HIV test, so further studies for exploring its hidden barriers are needed.

## Figures and Tables

**Table 1:** Individual and household characteristics of women aged 15–49 had a live birth in the last two years, Vietnam, Multiple Indicator Cluster Survey (MICS) 2014.

Characteristics	Individual and Household Characteristics of Women Aged 15–49	
	n	%
Age group		
15–19	80	5.4
20–34	1239	83.5
35–49	165	11.1
Ethnicity		
Minority	252	17.0
Kinh	1232	83.0
Educational level		
Primary or less	252	17.0
Lower secondary school	536	36.1
Upper secondary school and tertiary	696	46.9
Living area		
Urban	433	29.2
Rural	1051	70.8
Socioeconomic status		
Poorest	298	20.1
Poorer	292	19.7
Middle	297	20.0
Richer	318	21.4
Richest	279	18.8

**Table 2:** Human immunodeficiency virus (HIV) testing during antenatal care among women aged 15–49 who had a live birth in the last two years by different socioeconomic variables, Vietnam, MICS 2014.

Characteristics	Women Who Received HIV Testing during ANC		*p* value
	%	95%CI
Age group			0.1664
15–19	21.8	12.6–35.0	
20–34	29.8	26.3–33.6	
35–49	35.6	27.4–44.6	
Ethnicity			0.0000 ^***^
Minority	8.5	5.1–13.6	
Kinh	34.4	30.6–38.6	
Educational level			0.0000 ^***^
Primary or less	17.0	12.1–23.5	
Lower secondary school	25.3	20.9–30.3	
Upper secondary school and tertiary	38.4	33.6–43.4	
Living area			0.0000 ^***^
Urban	42.2	6.79–8.75	
Rural	25.0	20.8–29.7	
Socioeconomic status			0.0000 ^***^
Poorest	8.7	5.7–13.2	
Poorer	20.5	15.5–26.7	
Middle	34.9	28.0–42.6	
Richer	36.5	30.0–43.6	
Richest	50.1	42.4–57.8	

^***^: significant at 0.001; CI: Confidence interval.

**Table 3:** Concentration indices of HIV testing during antenatal care among women aged 15–49 years who had a live birth in the last two years by socioeconomic variables.

Characteristics	Women Who Received HIV Testing during ANC	
	CCI	SE
Age group		
15–19	0.4 ^**^	0.13
20–34	0.3 ^***^	0.03
35–49	0.3 ^***^	0.06
Ethnicity		
Minority	0.5 ^***^	0.11
Kinh	0.2 ^***^	0.03
Educational level		
Primary or less	0.4 ^***^	0.08
Lower secondary school	0.2 ^***^	0.05
Upper secondary school and tertiary	0.2 ^***^	0.03
Living area		
Urban	0.1 ^***^	0.04
Rural	0.3 ^***^	0.04

^*, **, ***^: significant at 0.05, 0.01 and 0.001; CCI: concentration index; SE: standard error; **ANC:** antenatal care.

**Table 4:** Multivariable logistic regression of factors associated with HIV testing during antenatal care among women aged 15–49 years who had a live birth in the last two years.

Characteristics	Women Who Received HIV Testing during ANC	
	OR	95%CI
Age group		
15–19	1	-
20–34	0.77	0.4–1.6
35–49	1.01	0.5–2.2
Ethnicity		
Minority	1	-
Kinh	2.52 ^**^	1.4–4.7
Educational level		
Primary or less	1	-
Lower secondary school	1.09	0.7–1.7
Upper secondary school and tertiary	1.37	0.9–2.2
Living area		
Urban	1	-
Rural	0.82	0.5–1.3
Socioeconomic status		
Poorest	1	-
Poorer	1.81	0.9–3.3
Middle	3.38 ^***^	1.9–6.2
Richer	3.30 ^***^	1.7–6.3
Richest	4.99 ^***^	2.4–10.3

^*, **, ***^: significant at 0.05, 0.01 and 0.001; OR: Odd ratio; CI: Confidence interval.
